# Effect of *TG5* and *LEP* polymorphisms on the productivity, chemical composition, and fatty acid profile of meat from Simmental bulls

**DOI:** 10.14202/vetworld.2023.1647-1654

**Published:** 2023-08-17

**Authors:** Irina Sycheva, Evgeniya Latynina, Azer Mamedov, Oksana Tsibizova, Yulia Kozak, Dmitriy Svistounov, Irina Bystrenina, Aleksandr Orishev

**Affiliations:** Department of Animal Science and Biology, Russian State Agrarian University - Moscow Timiryazev Agricultural Academy, Moscow, Russian Federation

**Keywords:** chemical composition, fatty acid, *leptin*, meat productivity, polymorphism, Simmental, *thyroglobulin*

## Abstract

**Background and Aim:**

Enhancing the nutritional and biological value of meat obtained from young surplus replacement animals of dual-purpose breeds is a critical objective in the livestock industry. This study aimed to investigate the impact of *thyroglobulin* (*TG5*, *c. −422C > T*) and *leptin* (*LEP*, *c. 239C > T*) polymorphisms on the productivity, chemical composition, and fatty acid (FA) profile of meat from Simmental bulls.

**Materials and Methods:**

A total of 26 Simmental bulls were genotyped for *TG5* (*c. −422C > T*) and *LEP* (*c. 239C > T*) polymorphisms and reared under the same fattening conditions. Controlled slaughter was conducted at 18 months of age. Subsequently, the experimental animals were evaluated to determine their slaughter traits and the chemical and FA composition of ground beef and the longissimus dorsi muscle.

**Results:**

The results showed that the *TG5* (*c. −422C > T*) polymorphism significantly (p < 0.05) affected the differentiation of bulls in terms of the synthesis of stearic acid, linolenic acid, and total polyunsaturated FAs, as well as the fat and dry matter content in the longissimus dorsi muscle. Conversely, the presence of the T allele in the *LEP* (*c. 239C > T*) polymorphism was associated with increased dry matter and fat in ground beef, carcass weight, and internal fat weight.

**Conclusion:**

The analysis of slaughter traits and the chemical and FA composition of meat from the Simmental bulls genotyped for the *TG5* and *LEP* genes revealed a genetic basis for the quantitative and qualitative characteristics of meat productivity. Thus, the genetic variability of bulls regarding the *LEP* and *TG5* genes can be used to improve the quantitative and qualitative indicators of meat productivity in Simmental cattle through marker-assisted selection.

## Introduction

More than 80% of domestic beef production in Russia comes from slaughtering dairy and dual-purpose cattle [1, 2]. Therefore, enhancing the nutritional and biological value of meat obtained from young surplus replacement animals of non-specialized breeds is an urgent task for the livestock industry. The most promising approach for efficient beef production involves intensive and long-growing cattle breeds with well-developed muscles and minimal subcutaneous fat deposition. Several breeds, including European continental breeds such as Charolais, Limousine, Chian, Blonde D’Aquitaine, and Maine, are gaining popularity due to their ability to produce heavier and leaner carcasses. These breeds are characterized by a late-maturing physiology, allowing them to build up (unlike early maturing breeds) muscle tissue up to 25–30 months, with an average daily gain of 1000–1300 g. However, the limited livestock numbers and poor acclimatization abilities of these breeds prevent mass distribution in the country. Consequently, interest in the Simmental breed has increased significantly in some regions. Simmental cattle are currently experiencing dynamic development for beef production worldwide and experts predict that their share in the total mass of beef cattle will continue to increase [3, 4].

Beef, derived from cattle muscle and adipose tissue, undergoes various physical and chemical processes. The level and intensity of these processes, and hence meat quality, are determined by genetic and paratypic factors, such as temperature, storage time, sex, and age of the animal [5, 6]. Red meat is an important source of high-quality protein and contains many essential nutrients. Rumen microflora in cattle hydrogenates unsaturated fatty acids (FAs) from their feed, resulting in relatively high levels of saturated FAs (SFAs) in beef compared to meat from monogastric animals. This phenomenon negatively affects beef demand, as nutritionists and doctors recommend reducing foods rich in SFAs in the diet [7, 8]. Nevertheless, the fat content and FA composition of beef remain important characteristics for both producers and consumers [9]. Many researchers have identified polymorphisms in the *leptin* (*LEP*) and *thyroglobulin* (*TG5*) genes as significant genetic factors that influence the intensity and characteristics of fat metabolism in the body [10, 11]. Thus, the *C422T* polymorphism occurs in the 5’-promoter region of the *TG5* gene [12]. This nucleotide substitution, C→T, promotes the appearance of two allelic variants, with carriers exhibiting noticeable differences in meat marbling scores, intramuscular fat content, and beef quality [13, 14]. Various genetic variations in the *LEP* gene are extensively used in marker-assisted selection (MAS) programs to predict carcass fat formation, body weight, and growth rate in cattle [15, 16]. Despite numerous associative analyses of the formation of meat productivity and beef quality, few studies have explored the FA profile of meat in relation to allelic variants of fat metabolism genes, particularly in dual-purpose cattle.

This study aimed to investigate the formation of meat productivity, chemical composition, and FA profile of beef in Simmental bulls based on *TG5* (*c. −422C > T*) and *LEP* (*c. 239C > T*) polymorphisms.

## Materials and Methods

### Ethical approval

The Local Ethics Committee of the Russian State Agrarian University-Moscow Timiryazev Agricultural Academy, Moscow, Russian Federation, approved the protocol of the present investigation (No. 2022/12 dated March 10, 2022). All animal studies were conducted following the ethical standards laid down in the 1964 Declaration of Helsinki and its later amendments. Every possible effort was made during the research to minimize animals’ distress and use fewer samples.

### Study period and location

The study was conducted from June 2021 to December 2022 at the test station of the “Dombarovskiy” Ltd. (Dombarovskiy District, Orenburg Region, Russia).

### Animals and sampling

Simmental bulls (n = 26) with different genotypes were reared under the same fattening conditions up to 18 months of age. Animal-controlled slaughter was conducted following State Standard R 34120–2017, “Cattle for slaughter, Beef and veal in carcasses, half carcasses, and quarters” [17]. A 400 g average sample of ground beef was taken from the left half-carcass, and a 200 g sample of the longissimus dorsi muscle was collected from the same half-carcass before deboning through a transverse cut between the 9^th^ and 11^th^ ribs.

The chemical and FA compositions of the ground beef and longissimus dorsi muscle were determined in the laboratory of the Department of Physiology, Morphology, and Biochemistry of Animals at the Russian State Agrarian University-Moscow Timiryazev Agricultural Academy. Dry matter content was determined by drying the samples in an oven at 100°C, while organic matter was determined by ashing the dried sample at 550°C [18]. The FA composition of the longissimus dorsi muscle was determined using gas-liquid chromatography with a Kristall-4000 Lux chromatograph (Russia).

### Genotyping

For genotyping based on the *TG5* (*c. −422C > T*) and *LEP* (*c. 239C > T*) gene polymorphisms, blood samples were taken from the jugular vein of the experimental bulls. DNA extraction was conducted using the DIAtom™ DNA Prep kit (IsoGeneLab, Moscow). GenePakPCRCore kits (IsoGeneLab) were used for polymerase chain reaction (PCR)-restriction fragment length polymorphism on a programmable “Tertsik” thermal cycler (DNA Technology, Russia). Specific primers (SPF “Litekh,” Russia), PCR conditions, and endonucleases were used in this study (Table-1).

**Table-1:** Primer sequence, PCR conditions, restriction enzymes used in genotyping.

SNP	Primer	PCR conditions	Restriction enzymes
*TG5 (c. -422C>T)*	F: 5’- ggg-gat-gac-tac-gag-tat-gac-tg-3’ R: 5’- gtg-aaa-atc-ttc-tgg-agg-ctg-ta-3’	Initial heating - at+94°C for 4 min; 35 cycles: denaturation - at+94°C for 60 s; annealing - at+62°C for 60 s; synthesis - at+72°C for 60 s; primerw extension - at+72°C for 4 min	*BstX2I*
*LEP (c. 239C>T)*	F: 5’- ggg-aag-ggc-aga-aag-ata-g-3’ R: 5’- cca-agc-tct-cca-agc-tct-c- 3’	Initial heating - at+95°C for 5 min; 30 cycles: denaturation - at+94°C for 30 s; annealing - at+62°C for 40 s; synthesis - at+72°C for 40 s; primer extension - at+72°C for 7 min	*Eco91I*

SNP=Single-nucleotide polymorphism, PCR=Polymerase chain reaction, *TG5=thyroglobulin*, *LEP=leptin*

Product splitting was conducted at 37°C and genotypes were identified by gel electrophoresis with visualization under ultraviolet light.

### Statistical analysis

The effects of genotype on the studied traits were analyzed using the least-squares method with the general linear model procedure of Statistica 10.0 software (“Stat Soft Inc.,” USA).

Model used:

Y_ij_=μ+A_i_+e_ij_,

Where, Y_ij_ – represents the studied traits, μ – is the overall mean, A_i_ – is the fixed effect of the TG5 and LEP genotype (1, 2, 3), and e_ij_ – is a random error.

The significance of inter-genotype differences was assessed using a posteriori Fisher’s criterion (F-test). p ≤ 0.05 was considered statistically significant.

## Results

The polymorphism of the *LEP* gene significantly impacted the variability of the slaughter indicators in Simmental bulls (Table-2). Heterozygous individuals showed a significant advantage in preslaughter live weight by 21.8 kg (4.15%; p < 0.05), carcass weight by 17.8 kg (6.16%; p < 0.05), and slaughter weight by 18.2 kg (6.01%; p < 0.05) compared to carriers of the *LEP^CC^* genotype. The greatest internal fat development was observed during the slaughter of bulls homozygous for the thymine allele, which exceeded alternative homozygous genotypes in internal fat weight and yield by 2.7 kg (19.71%; p < 0.05) and 0.5%, respectively. Heterozygous animals differed in their maximum indicators of carcass and slaughter yields, with the proportion of deposited fat at a minimum level. This finding indicates the relative late maturity of carriers of the *LEP^CT^* genotype. The T allele in the *LEP* (*c. 239C > T*) polymorphism was associated with increased live and carcass weight in Simmental bulls. However, when this allele was homogenized, fat accumulation significantly intensified with a slight decrease in weight growth and carcass weight relative to heterozygous peers.

**Table-2:** Slaughter traits in Simmental bulls depending on *LEP (c. 239C>T)* polymorphism (M ± m).

Indicator	*LEP (c. 239C>T)* genotype		

	CC (n=12)	CT (n=7)	TT (n=7)
Pre-slaughter live weight, kg	525.2 ± 5.77^b^	547.0 ± 8.24^a^	537.4 ± 6.60^a,b^
Carcass weight, kg	289.1 ± 5.53^b^	306.9 ± 6.70^a^	298.6 ± 4.80^a,b^
Carcass yield, %	55.1 ± 0.94	56.1 ± 1.03	55.6 ± 0.54
Internal fat weight, kg	13.7 ± 0.87^b^	14.0 ± 0.90^a,b^	16.4 ± 0.48^a^
Internal fat yield, %	2.6 ± 0.18	2.5 ± 0.13	3.1 ± 0.10
Slaughter weight, kg	302.7 ± 5.04^b^	320.9 ± 7.15^a^	315.0 ± 4.60^a,b^
Slaughter yield, %	57.7 ± 0.87	58.7 ± 1.01	58.6 ± 0.49

a,b Values with different superscripts are significantly different at p < 0.05. *LEP=Leptin*, CC=genotype CC, CT=genotype CT, TT=genotype TT, n=number of animals

No significant differences existed in slaughter traits between the two available genotypes in the *TG5* gene polymorphism of Simmental bulls (Table-3). Homozygous young animals were superior in carcass weight and yield by 6.2 kg (2.11%; p = 0.40) and 1.3% (p = 0.21), respectively. Heterozygous individuals had an advantage in internal fat development, outperforming their peers in fat weight and yield by 0.7 kg (1.3%; p = 0.64) and 0.7% (p = 0.67), respectively.

**Table-3:** Slaughter traits in Simmental bulls depending on *TG5 (c. -422C>T)* polymorphism (M ± m).

Indicator	*TG5 (c. -422C>T)* genotype	

	CC (n=11)	CT (n=15)
Pre-slaughter live weight, kg	533.3 ± 4.58	535.2 ± 6.52
Carcass weight, kg	300.0 ± 5.93	293.8 ± 4.48
Carcass yield, %	56.2 ± 0.94	54.9 ± 0.57
Internal fat weight, kg	14.1 ± 0.64	14.8 ± 0.79
Internal fat yield, %	2.6 ± 0.12	2.8 ± 0.15
Slaughter weight, kg	314.1 ± 5.98	308.6 ± 4.30
Slaughter yield, %	58.9 ± 0.95	57.7 ± 0.49

*TG=Thyroglobulin*, CC=genotype CC, CT=genotype CT, n=number of animals

Differentiation of bulls according to the *LEP* (*c. 239C > T*) polymorphism affected the chemical composition of the average ground beef sample (Table-4). The T allele was associated with increased dry matter content in beef. Bulls homozygous for the thymine allele outperformed their peers with the CC-genotype by 2.67% (p < 0.01) and heterozygous carriers by 1.79% (p < 0.05). This advantage was mainly due to the intensive fat accumulation in the carcass flesh. Moreover, significant differences (2.99%; p < 0.01) in fat content were observed between the homozygous representatives of Simmentals. Heterozygous bulls exhibited an intermediate intensity of fat deposition in ground beef. However, the variability in protein content in the average ground beef sample was low and did not depend on the *LEP* gene polymorphism.

**Table-4:** Chemical composition of ground beef and longissimus dorsi muscle in Simmental bulls depending on *LEP (c. 239C>T)* polymorphism (M ± m), %.

Indicator	*LEP (c. 239C>T)* genotype		

	CC (n=12)	CT (n=7)	TT (n=7)
Ground beef			
Dry matter	30.38 ± 0.520^a^	31.26 ± 0.208^b^	33.05 ± 0.581^c^
Fat	9.89 ± 0.744^a^	11.00 ± 0.565^a,b^	12.88 ± 0.788^b^
Protein	19.55 ± 0.320	19.33 ± 0.472	19.24 ± 0,388
Ash	0.94 ± 0.009	0.93 ± 0.012	0.93 ± 0.013
Longissimus dorsi muscle			
Dry matter	23.46 ± 0.240	23.22 ± 0.387	23.41 ± 0.260
Fat	1.68 ± 0.192	1.73 ± 0.166	2.14 ± 0.313
Protein	20.81 ± 0.243	20.52 ± 0.244	20.32 ± 0.200
Ash	0.97 ± 0.015	0.97 ± 0.017	0.95 ± 0.008

^a,b,c^Values with different superscripts are significantly different at p < 0.05. *LEP=Leptin*, CC=genotype CC, CT=genotype CT, TT=genotype TT, n=number of animals

The chemical composition of the longissimus dorsi muscle was influenced to a lesser degree by *LEP* (*c. 239C > T*) polymorphism. There was a tendency toward increased fat synthesis in muscle tissue in TT-homozygotes relative to CC-genotype by 0.46% (p = 0.16) and heterozygous individuals by 0.41% (p = 0.26). Conversely, homozygotes for cytosine tended toward more intensive protein synthesis, providing them with a 0.29%–0.49% (p > 0.05) advantage over their peers.

The advantage of heterozygous individuals in total nutrient accumulation in carcass flesh was observed when the bulls were genotyped for the *TG5* gene; however, intergroup differences were not significant (Table-5). The superiority of CT-genotype in fat and protein content was 0.77% (p = 0.44) and 0.38% (p = 0.39). Consequently, ground beef from *TG5^CC^* homozygous young animals contained the least amount of dry matter, which was 1.16% inferior to that of their heterozygous peers (p = 0.11).

**Table-5:** Chemical composition of ground beef and longissimus dorsi muscle in Simmental bulls depending on *TG5 (c. -422C>T)* polymorphism (M ± m), %.

Indicator	*TG5 (c. -422C>T)* genotype	

	CC (n=11)	CT (n=15)
Ground beef		
Dry matter	30.67 ± 0.401	31.83 ± 0.522
Fat	10.55 ± 0.611	11.32 ± 0.715
Protein	19.19 ± 0.328	19.57 ± 0.283
Ash	0.93 ± 0.011	0.94 ± 0.007
Longissimus dorsi muscle		
Dry matter	22.81 ± 0.194^a^	23.81 ± 0.175^b^
Fat	1.28 ± 0.101^a^	2.22 ± 0.146^b^
Protein	20.56 ± 0.169	20.62 ± 0.218
Ash	0.96 ± 0.011	0.97 ± 0.012

^a,b^Values with different superscripts are significantly different at p < 0.001. *TG=Thyroglobulin*, CC=genotype CC, CT=genotype CT, n=number of animals

The *TG5* (*c. −422C > T*) polymorphism had a more significant effect on the chemical composition of longissimus dorsi muscle compared to *LEP* gene. In particular, a highly significant superiority (by 0.94%; p < 0.001) of the heterozygous genotype was observed in fat accumulation in muscle tissue. There were less noticeable intergroup differences in protein synthesis. Thus, the muscles of heterozygous bulls contained 1.0% (p < 0.001) drier matter compared to their homozygous peers.

The analysis of the FA profile of the longissimus dorsi muscle in Simmental bulls indicated a significant association between the *TG5* (*c. −422C > T*) polymorphism and the stearic (C_18:0_) acid content (Table-6). Carriers of the homozygous *TG5^CC^* genotype outperformed their heterozygous peers by 0.85% (p < 0.05) regarding the amount of this FA in the lipid structure. Intergroup differences were not significant for other SFAs, although heterozygous individuals had a trend-level advantage in the synthesis of myristic by 0.11% (p = 0.26) and palmitic by 0.55% (p = 0.58).

**Table-6:** Fatty acid composition of meat from Simmental bulls depending on *TG5 (c. -422C>T)* polymorphism (M ± m), %.

Fatty acid	*TG5 (c. -422C>T)* genotype	

	CC (n=11)	CT (n=15)
Saturated fatty acids (SFA)		
Myristic (C_14:0_)	2.54 ± 0.068	2.65 ± 0.068
Palmitic (C_16:0_)	25.64 ± 0.759	26.19 ± 0.628
Stearic (C_18:0_)	22.61 ± 0.254^b^	21.76 ± 0.238^a^
Monounsaturated fatty acids (MUFA)		
Myristoleic (C_14:1_)	0.54 ± 0.055	0.61 ± 0.038
Palmitoleic (C_16:1_)	3.47 ± 0.078	3.33 ± 0.097
Oleic (C_18:1_)	38.95 ± 0.675	38.75 ± 0.443
Polyunsaturated fatty acids (PUFA)		
Linoleic (C_18:2_)	4.21 ± 0.145	4.42 ± 0.118
Linolenic (C_18:3_)	0.20 ± 0.023^a^	0.27 ± 0.023^b^
Arachidonic (C_20:4_)	1.84 ± 0.093	2.02 ± 0.069

^a,b^Values with different superscripts are significantly different at p < 0.05. *TG=Thyroglobulin*, CC=genotype CC, CT=genotype CT, n=number of animals

The high content of monounsaturated FAs (MUFAs) in the lipid structure is associated with a low melting point of intramuscular fat. It positively affects the organoleptic characteristics of beef, particularly its taste and tenderness. The most important and representative MUFA in beef fat is oleic acid (C_18:1_), which varies within a narrow range of 38.75%–38.95% during genotyping for the *TG5* gene. The intergroup differences were also insignificant for the other MUFAs. Thus, an advantage in the myristoleic (C14:1) acid content was observed among heterozygous individuals, amounting to 0.07% (p = 0.30), while homozygous individuals showed a 0.14% advantage (p = 0.29) in palmitoleic acid.

The content of specific polyunsaturated FAs was largely determined by the allelic variant of the *TG5* gene due to the low variability of SFAs and MUFAs in meat, depending on the *TG5* (*c. −422C > T*) polymorphism. Heterozygous bulls exhibited a significant advantage over their homozygous peers in the amount of linolenic (C_18:3_) acid by 0.07% (p < 0.05). In addition, they had a trend-level advantage in the synthesis of linoleic (0.21%; p = 0.26) and arachidonic (0.18%; p = 0.12) acids.

Our studies showed that the *LEP* (*c. 239C > T*) polymorphism had a less noticeable effect on the FA profile of beef from Simmental bulls (Table-7). However, some features of FA synthesis were observed due to the genotype of the *LEP* gene. Notably, the meat from bulls homozygous for cytosine had the highest content of stearic and palmitic acids, outperforming their peers by 0.42%–0.77% (p > 0.05) and 0.53%–0.88% (p > 0.05), respectively. Conversely, homozygotes for thymine exhibited an increased concentration of myristic (C_14:0_) FA, with an advantage of 0.05%–0.10% (p > 0.05) compared to other genotypes for the *LEP* gene.

**Table-7:** Fatty acid composition of meat from Simmental bulls depending on *LEP (c. 239C>T)* polymorphism (M ± m), %.

Fatty acid	*LEP (c. 239C>T)* genotype		

	CC (n=12)	CT (n=7)	TT (n=7)
Saturated fatty acids (SFA)			
Myristic (C_14:0_)	2.56 ± 0.074	2.61 ± 0.087	2.66 ± 0.107
Palmitic (C_16:0_)	26.34 ± 0.665	25.81 ± 1.185	25.46 ± 0.806
Stearic (C_18:0_)	22.44 ± 0.239	21.67 ± 0.454	22.02 ± 0.337
Monounsaturated fatty acids (MUFA)			
Myristoleic (C_14:1_)	0.56 ± 0.049	0.60 ± 0.056	0.60 ± 0.070
Palmitoleic (C_16:1_)	3.35 ± 0.111	3.52 ± 0.097	3.33 ± 0.119
Oleic (C_18:1_)	38.31 ± 0.538	39.13 ± 0.874	39.44 ± 0.596
Polyunsaturated fatty acids (PUFA)			
Linoleic (C_18:2_)	4.30 ± 0.129	4.41 ± 0.203	4.30 ± 0.191
Linolenic (C_18:3_)	0.23 ± 0.023	0.23 ± 0.033	0.27 ± 0.040
Arachidonic (C_20:4_)	1.92 ± 0.096	2.00 ± 0.131	1.92 ± 0.068

*LEP=Leptin*, CC=genotype CC, CT=genotype CT, TT=genotype TT, n=number of animals

Homozygous and heterozygous carriers of the T allele tend to increase MUFA accumulation in muscle tissue. They surpassed their peers in myristoleic and oleic acid content by 0.04% and 0.82%–1.13%, respectively.

Meat from heterozygous bulls for the *LEP* (*c. 239C > T*) polymorphism was characterized by the best composition of polyunsaturated FAs. Carriers of the *LEP^CT^* genotype had an advantage in the proportion of linoleic and arachidonic acid by 0.11% and 0.08%, respectively, relative to their homozygous peers. Conversely, the *LEP^TT^* genotype was associated with an increased (by 0.04%) linolenic (C_18:3_) acid content.

Integral indicators of lipid metabolism largely characterize the meat quality of Simmental bulls. Thus, the homozygous *TG5^CC^* young animals slightly exceeded the SFA (0.19%) and MUFA (0.27%) content of their heterozygous peers (Table-8). In turn, carrying the heterozygous *TG5^CT^* genotype led to a significant advantage (0.47%; p < 0.05) in polyunsaturated FA synthesis.

**Table-8:** Characteristics of lipids in the meat of Simmental bulls depending on TG5 (c. -422C>T) polymorphism (M ± m).

Indicator	*TG5 (c. -422C>T)* genotype	

	CC	CT
SFA amount, %	50.79 ± 0.776	50.60 ± 0.461
MUFA amount, %	42.96 ± 0.647	42.69 ± 0.427
PUFA amount, %	6.24 ± 0.208^a^	6.71 ± 0.113^b^
UFA: SFA ratio, units	0.97 ± 0.030	0.98 ± 0.018
PUFA: SFA ratio, units	0.12 ± 0.006	0.13 ± 0.003

^a,b^Values with different superscripts are significantly different at p < 0.05. *TG=Thyroglobulin*, CC=genotype CC, CT=genotype CT, SFA=saturated fatty acids, MUFA=monounsaturated fatty acids, PUFA=polyunsaturated fatty acids, UFA=unsaturated fatty acids

Genotyping for the *LEP* (*c. 239C > T*) polymorphism also indicated the superiority (0.16–0.21%; p > 0.05) of heterozygous carriers in polyunsaturated FA synthesis (Table-9). Meat from young animals homozygous for cytosine was rich in SFA, with an advantage of 1.20%–1.24% (p > 0.05) over individuals with the T allele. This resulted in the minimum ratio of unsaturated to SFAs in representatives of the *LEP^CC^* genotype.

**Table-9:** Characteristics of lipids in the meat of Simmental bulls depending on *LEP (c. 239C>T)* polymorphism (M ± m)

Indicator	*LEP (c. 239C>T)* genotype		

	CC	CT	TT
SFA amount, %	51.34 ± 0.628	50.10 ± 0.805	50.14 ± 0.728
MUFA amount, %	42.22 ± 0.515	43.25 ± 0.783	43.37 ± 0.624
PUFA amount, %	6.44 ± 0.178	6.65 ± 0.153	6.49 ± 0.287
UFA: SFA ratio, units	0.95 ± 0.024	1.00 ± 0.031	1.00 ± 0.029
PUFA: SFA ratio, units	0.13 ± 0.005	0.13 ± 0.004	0.13 ± 0.007

*LEP=Leptin*, CC=genotype CC, CT=genotype CT, TT=genotype TT, SFA=saturated fatty acids, MUFA=monounsaturated fatty acids, PUFA=polyunsaturated fatty acids, UFA=unsaturated fatty acids.

## Discussion

Identifying functional polymorphisms in genes associated with meat productivity and beef quality is intended to optimize herd completion with high-value animals and accelerate selection and breeding efforts [19, 20]. Karisa *et al*. [21] reported that the effect of a specific quantitative trait locus on the variability of selected traits in beef cattle could reach 19.7%. Kashi *et al*. [22] stated that increasing the frequency of carriers of “desirable” genotypes could lead to an annual selection effect of 15%–30% in cattle herds.

In our research, the variability of slaughter indicators and the chemical composition of carcass flesh in Simmental bulls, depending on the genotypes for the *LEP* and *TG5* genes, showed significant differences in the degree of determination of phenotypic variability of traits using the studied polymorphisms. For instance, the *LEP* (*c. 239C > T*) single-nucleotide polymorphism determined the variability of preslaughter live weight by 19.36% (p = 0.08), carcass weight by 17.30% (p = 0.11), internal fat weight by 14.63% (p = 0.16), internal fat yield by 12.65% (p = 0.21), slaughter weight by 20.12% (p = 0.08), fat content in ground beef by 26.01% (p < 0.05), and dry matter content by 38.02% (p < 0.01). Our findings were consistent with the results of Ardicli *et al*. [23], who confirmed the significant effect of the *LEP* gene on slaughter traits in Simmental animals. The contribution of the *LEP* gene polymorphism to the variability in the chemical composition of muscle tissue was slightly smaller. In contrast, the substitution C→T in the nucleotide sequence of the *TG5* gene did not result in significant changes in slaughter indicators and the chemical composition of carcass flesh. Due to the low variability in protein content, differences in the chemical composition of ground beef were mainly due to the unequal intensity of fat deposition in carcasses from various genotypes of bulls when they were differentiated according to the *LEP* gene polymorphism, leading to the highest variability in the protein and fat ratio in carcass flesh (1:0.51–0.67). Conversely, the nucleotide substitution in the *TG5* gene provided a variation of this parameter within 1:0.55–0.58.

The *TG5* (*c. −422C > T*) polymorphism significantly (p < 0.001) explained the variability in fat content by 49.94% and dry matter content by 37.64% in the longissimus dorsi muscle. However, the content of major nutrients in ground beef showed low determination using the allelic structure of the *TG5* gene, mainly due to the unequal distribution of alternative alleles in the herd. This allelic frequency limits selection for creating the optimal genetic structure in the population, considering the *TG5* gene [24]. Nevertheless, Miroshnikov *et al*. [25] reported sufficient genetic diversity of domestic breeding herds of beef cattle as the main condition for effective MAS.

Shevkhuzhev *et al*. [26] recommended utilizing additive variability to increase the desired homozygosity and genetic potential in populations with purebred breeding of animals. Bennett *et al*. [27] proposed the intensive use of sires heterozygous for the *CSN1S1 × TG5* genetic complex to increase the frequency of desirable alleles in the gene pool of an improved herd. Therefore, despite long-term purebred breeding, the Simmental breed retains significant genetic diversity that can further contribute to improvement through intrabreed resources [28].

Analysis of the chemical composition of the longissimus dorsi muscle in Simmental bulls showed a significantly greater determination of nutrient content depending on *TG5* (*c. −422C > T*) polymorphism compared to the allelic profile for the *LEP* gene. A similar conclusion was reached by Sedykh *et al*. [29], who observed a significant (p < 0.05) superiority of *TG5^TT^* genotype carriers in terms of fat content in muscle tissue relative to their peers in Hereford bulls. However, they did not find significant differences between Hereford bulls of various genotypes regarding the *LEP* gene.

De Smet *et al*. [30] reported that intensive fat deposition in animals leads to an increased synthesis of SFAs and MUFAs, resulting in a decrease in the proportion of polyunsaturated FAs in muscle tissue. In addition, Lapshina *et al*. [31] found that high intensity of weight growth negatively affected the quality of beef lipids. This observation is supported by our study, as more massive carriers of the T allele had the highest MUFA content for the *LEP* gene polymorphism, while bulls with the *TG5^CT^* genotype, characterized by the smallest carcass weight, had the maximum amount of polyunsaturated FAs.

Our study showed that the *TG5* (*c. −422C > T*) polymorphism had a greater effect on the lipid composition of muscle tissue in cattle compared to the nucleotide substitution in the *LEP* gene. Specifically, differentiating Simmental bulls using the *TG5* gene significantly determined the variability in the stearic acid (by 19.74%; p < 0.05), linolenic acid (by 14.69%; p < 0.05), and polyunsaturated FA (by 15.41%; p < 0.05) content. Carriers of the T allele showed increased synthesis of polyunsaturated FAs. A similar significant effect of *TG5* gene polymorphism on the FA composition of blood serum was found in Hereford cattle [32]. Considering that a high content of unsaturated FAs is associated with juiciness [33] and is considered the main factor in the appearance of flavor in cooked meat [34], the T allele can be regarded as “desirable” in Simmental cattle selection for the biological and culinary value of beef.

## Conclusion

The analysis of slaughter traits, chemical composition, and FA profile of meat from Simmental bulls, genotyped for *TG5* and *LEP* genes, revealed the genetic determination of the quantitative and qualitative characteristics of meat productivity. The *TG5* (*c. –422C > T*) polymorphism significantly (p < 0.05) affected the differentiation of bulls in terms of the synthesis of stearic acid, linolenic acid, and total polyunsaturated FAs, as well as the fat and dry matter content in the longissimus dorsi muscle. Similarly, the T allele in the *LEP* (*c. 239C > T*) polymorphism was associated with increased levels of dry matter and fat in ground beef, carcass weight, and internal fat weight. Thus, the genetic variability of bulls in terms of the *LEP* and *TG5* genes can be used to improve the quantitative and qualitative indicators of meat productivity in Simmental cattle using MAS.

## Authors’ Contributions

IS, EL, AM, OT, YK, DS, IB, and AO: Contributed equally to the experimentation. IS and EL: Wrote and edited the manuscript. AM, OT, and YK: Equally designed and conducted the experiment. DS, IB, and AO: Studied scientific literature about the topic. All authors have read, reviewed, and approved the final manuscript.
